# Stem cells from human exfoliated deciduous teeth attenuate mechanical allodynia in mice through distinct from the siglec-9/MCP-1-mediated tissue-repairing mechanism

**DOI:** 10.1038/s41598-021-99585-2

**Published:** 2021-10-08

**Authors:** Yoshinori Hayashi, Hiroki Kato, Kazuaki Nonaka, Hiroshi Nakanishi

**Affiliations:** 1 Department of Physiology, Nihon University School of Dentistry, Tokyo, 101-8310 Japan; 2 Faculty of Dental Science, Department of Aging Science and Pharmacology, Kyushu University, Fukuoka, 812-8582 Japan; 3 Department of Molecular Cell Biology and Oral Anatomy, Division of Oral Biological Sciences, Graduate School of Dental Science, Kyushu University, Maidashi 3-1-1, Higashi-Ku, Fukuoka, 812-8582 Japan; 4 Section of Oral Medicine for Children, Division of Oral Health, Growth and Development, Faculty of Dental Science, Kyushu University, Fukuoka, 812-8582 Japan; 5 School of Health Sciences at Fukuoka, International University of Health and Welfare, Okawa, Fukuoka 831-8501 Japan; 6 Department of Pharmacology, Faculty of Pharmacy, Yasuda Women’s University, Hiroshima, 731-0153 Japan

**Keywords:** Sensory processing, Multipotent stem cells

## Abstract

The effects of stem cells from human exfoliated deciduous teeth (SHED) on mechanical allodynia were examined in mice. A single intravenous injection of SHED and conditioned medium from SHED (SHED-CM) through the left external jugular vein significantly reversed the established mechanical allodynia induced by spinal nerve transection at 6 days after injection. SHED or SHED-CM significantly decreased the mean numbers of activating transcription factor 3-positive neurons and macrophages in the ipsilateral side of the dorsal root ganglion (DRG) at 20 days after spinal nerve transection. SHED or SHED-CM also suppressed activation of microglia and astrocytes in the ipsilateral side of the dorsal spinal cord. A single intravenous injection of secreted ectodomain of sialic acid-binding Ig-like lectin-9 and monocyte chemoattractant protein-1 had no effect on the established mechanical allodynia, whereas a single intravenous injection of protein component(s) contained in SHED-CM with molecular weight of between 30 and 50 kDa reversed the pain. Therefore, it may be concluded that protein component(s) with molecular mass of 30–50 kDa secreted by SHED could protect and/or repair DRG neurons damaged by nerve transection, thereby ameliorating mechanical allodynia.

## Introduction

Stem cells from human exfoliated deciduous teeth (SHED) are thought to originate from the cranial neural crest with high self-renewal ability residing within the perivascular niche of the dental pulp^1,2^. Previous studies showed that SHED differentiated into functional neurons and oligodendrocytes under appropriate conditions and have immunomodulatory and regenerative activities^3–5^. Transplantation of SHED in the injured site promoted functional recovery from various acute and chronic insults of the central nervous system through paracrine mechanisms that activate endogenous tissue-repairing activities^6–9^. Besides SHED, the conditioned serum-free medium from SHED (SHED-CM) also exhibited tissue-repairing activities in an experimental autoimmune encephalomyelitis (EAE) mouse model of multiple sclerosis^10^ and a rat model of spinal cord injury^11,12^. Moreover, SHED-CM improved cognitive functions in a mouse model of Alzheimer’s disease^13^ and motor functions in a rat model of Parkinson’s disease^14^. More importantly, secreted ectodomain of sialic acid-binding Ig-like lectin-9 (ED-Siglec-9) and monocyte chemoattractant protein-1 (MCP-1) have been identified as a novel set of M2 inducers contained in SHED-CM to improve spinal cord injury^11^. A single intravenous injection of ED-Siglec-9 alone could improve the clinical symptoms of EAE^10^, probably because the level of endogenous MCP-1 is upregulated at the peak of EAE through direct modulation of M2 macrophage polarization^15^. ED-Siglec-9 is a major component of SHED-CM, while it is barely detectable in bone marrow mesenchymal stem cell (BMMSC)-CM. Therefore, it is considered that mesenchymal stem cell-derived secreted factors directly convert the proinflammatory conditions prevalent in the damaged neurons to tissue-repairing ones by modulating the microglia/macrophage phenotype.

Neuropathic pain is a debilitating symptom, which is caused by tissue or nerve damage. Neuropathic pain is characterized by severe pain produced by light touch. Accumulating evidence indicates that microglia in the spinal dorsal horn are highly activated following peripheral nerve injury^16,17^. Notably, daily administration of morphine, known as a powerful painkiller, leads to the activation of spinal microglia and causes hyperalgesia^18^. Currently available analgesics are not sufficiently treated neuropathic pain. A recent study revealed that macrophages in the dorsal root ganglion (DRG) are also required in the initiation and maintenance of neuropathic pain^19^. Thus, the importance of activated macrophages/microglia has been implicated in intractable neuropathic pain. Recent studies clarified the therapeutic significance of SHED in neuropathic pain caused by infraorbital nerve injury or diabetics in rats^12,20,21^. Analgesic mechanisms by SHED administration are revealed that SHED suppresses the upregulation of transient receptor potential vanilloid type 1 (TRPV1) in trigeminal ganglion neurons following infraorbital nerve injury^20^. However, SHED-mediated anti-allodynic effects are not fully understood. In the current study, we aimed to investigate the effects of SHED or SHED-CM on activated macrophage/microglia during neuropathic pain.

## Results

### Anti-allodynic effect of a single intravenous injection of SHED

**Figure 1:**
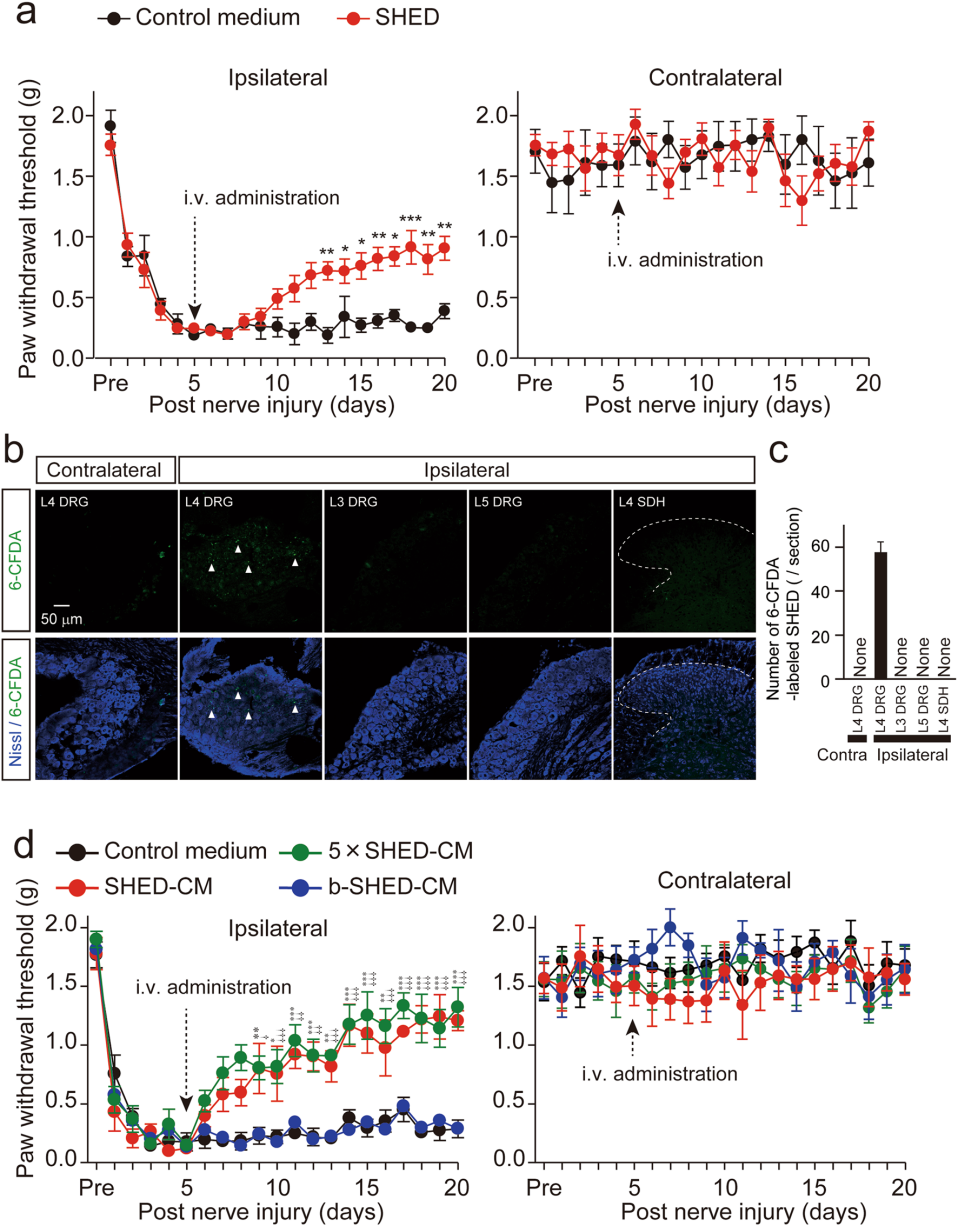
The effects of SHED and SHED-CM administration on the development of mechanical allodynia. (**a**) The effects of SHED administration on the established nerve injury-induced mechanical allodynia. SHED administration through the external jugular vein significantly reversed the established mechanical allodynia, whereas control medium showed no effect on the mean PWT. Data represent the mean ± SEM. n = 5 (control medium); n = 7 (SHED), two-way repeated-measures ANOVA post hoc Bonferroni’s test (*p < 0.05, **p < 0.01, ***p < 0.001, vs. control medium). (**b**) Localization of 6-CFDA-labeled SHED in the DRG following intrajuglar injection. 6-CFDA-labeled SHEDs were detected in the ipsilateral side of L4 DRG, but not in L3 or L5 DRG or L4 spinal dorsal horn. Arrowheads indicate 6-CFDA positive cells. Broken lines indicate the border of white and gray matter in the spinal dorsal horn. Scale bars = 50 μm. (**c**) The column represents the mean number of 6-CFDA-labeled SHED. Data represent the mean ± SEM. n = 6. (**d**) The effect of SHED-CM administration on the established nerve injury-induced mechanical allodynia. SHED-CM administration through the external jugular vein significantly reversed the established mechanical allodynia, whereas boiled SHED-CM (b-SHED-CM) showed no effect on the mean PWT. Data represent the mean ± SEM. n = 6 (Control medium); n = 5 (SHED-CM); n = 6 (5× SHED-CM); n = 5 (b-SHED-CM), two-way repeated-measures ANOVA post hoc Dunnet’s test (*p < 0.05, **p < 0.01, ***p < 0.001, Control medium vs. SHED-CM, ^†^*p* < 0.05, ^††^*p* < 0.01, ^†††^*p* < 0.001, Control medium vs. 5× SHED-CM).

A significant decrease in the paw withdrawal threshold (PWT) was observed from 1 day after peripheral nerve injury (PNI) in control medium-administered group and the reduction of the PWT following PNI was continued throughout the experimental period (Fig. 1a, control medium, n = 5). No change was observed in the contralateral side of the hind paw (Fig. 1a). To evaluate the anti-allodynic effect of SHED, a single administration of SHED through the left external jugular vein was conducted 5 days after PNI. A significant PWT recovery was observed from day 8 after intrajuglar administration of SHED (Fig. 1a, SHED, n = 7).

### SHED accumulates in the injured DRG following intravenous injection

In order to examine the localization of SHED after an intravenous administration, 6-carboxyfluorescein diacetate (CFDA)-labeled SHED was injected into the left external jugular vein 2 days after nerve injury. 6-CFDA-labeled SHED was detected in the ipsilateral side but not contralateral of L4 DRG at 3 days after injection, but not either the ipsilateral L3 or L5 DRG or L4 spinal dorsal horn (Fig. 1b,c).

### Anti-allodynic effect of protein components secreted from SHED

Next, the effect of a single intravenous injection of SHED-CM was examined at 5 days after PNI. SHED-CM administration significantly reversed the established mechanical allodynia at 4 days after injection (Fig. 1d, SHED-CM). Five-fold concentrated SHED-CM (5× SHED-CM) had almost the same anti-allodynic effect as SHED-CM (Fig. 1d, 5× SHED-CM). In contrast, boiled SHED-CM had no significant effect on the mean PWT (Fig. 1d, b-SHED-CM), suggesting that SHED-CM includes protein components. No change was observed in the contralateral side of the hind paw (Fig. 1d).

### Inhibitory effects of SHED or SHED-CM on the induction of activating transcription factor 3 (ATF3) and the accumulation of macrophages in the DRG

**Figure 2:**
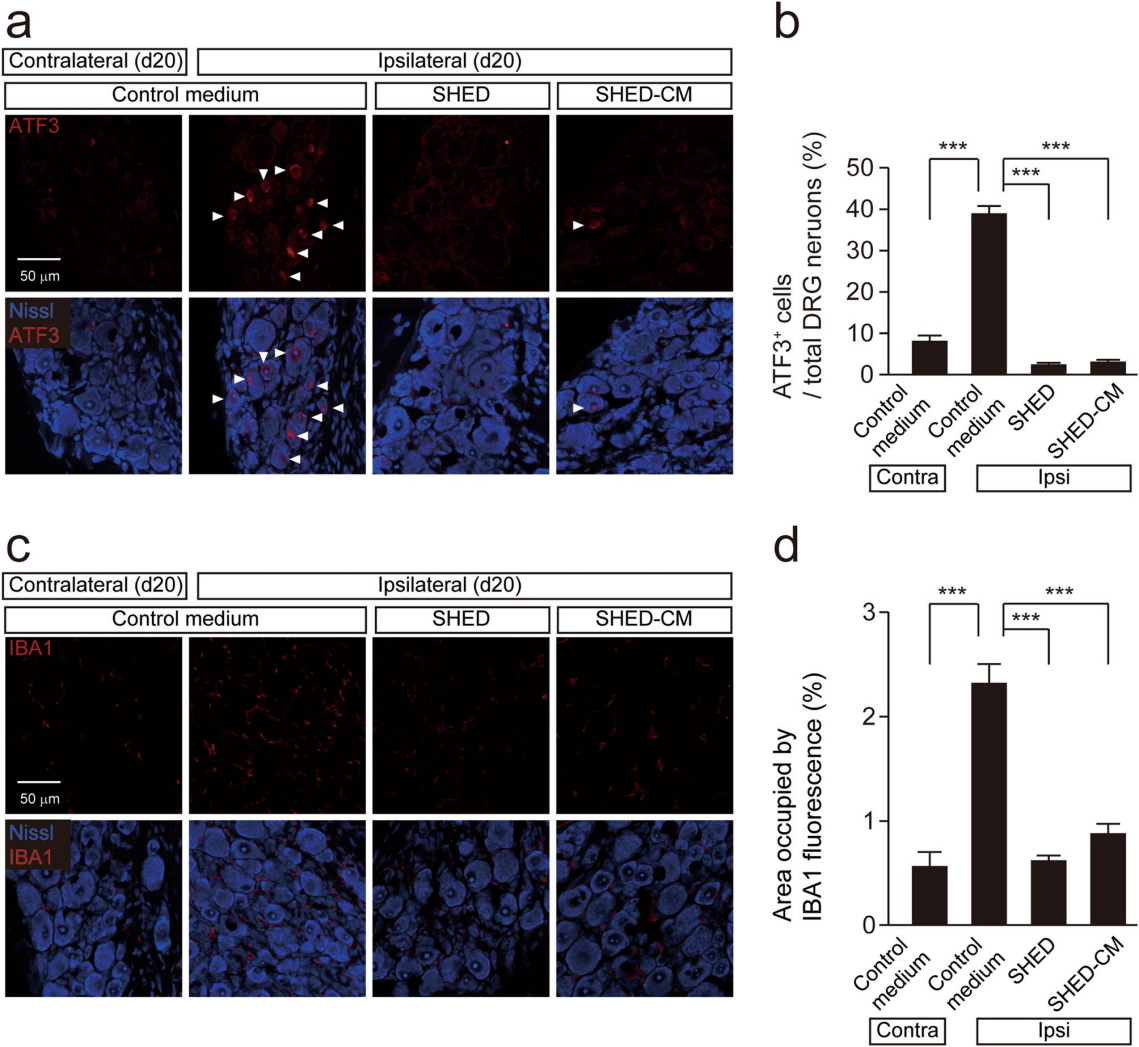
The inhibitory effect of SHED and SHED-CM on the AFT3 expression and macrophage accumulation in the ipsilateral side of DRG after nerve injury. (**a**) The image showing ATF3 (red) and Nissl (blue) immunofluorescence in the DRG 20 days after peripheral nerve injury (PNI). Arrowheads indicate ATF3 positive neurons. (**b**) The mean number of ATF3 positive neurons in the ipsilateral side of DRG following PNI. Data represent the mean ± SEM. n = 5 (control medium, contralateral: 222 ATF3 positive neurons/2820 Nissl positive neurons, ipsilateral: 546 ATF3 positive neurons/1386 Nissl positive neurons); n = 7 (SHED, 60 ATF3 positive neurons/2361 Nissl positive neurons); n = 5 (SHED-CM, 57 ATF3 positive neurons/1752 Nissl positive neurons), one-way ANOVA post hoc Tukey’s test. Asterisks indicate a statistically significant difference between the values (***p < 0.001). (**c**) The image showing IBA1 (red) and Nissl (blue) immunofluorescence in the DRG 20 days after PNI. (**d**) The mean area occupied by IBA1 fluorescence in the ipsilateral side of DRG following PNI. Data represent the mean ± SEM. n = 5 (control medium); n = 7 (SHED); n = 5 (SHED-CM,), one-way ANOVA post hoc Tukey’s test. Asterisks indicate a statistically significant difference between the values (*p < 0.05, **p < 0.01, ***p < 0.001).

Peripheral nerve injury induces the expression of ATF3, a marker of neuronal injury^22^, and accumulation of macrophages in the injured DRG^19^. Therefore, the effects of intravenous administration of SHED or SHED-CM on the expression of ATF3 and the accumulation of macrophages in the DRG were investigated.The mean number of ATF3-positive neurons was significantly increased in the ipsilateral side of the L4 DRG at 20 days after PNI (Fig. 2a,b). Intravenous administration of SHED or SHED-CM significantly reduced the mean number of ATF3-positive neurons (Fig. 2a,b). Marked accumulation of macrophages was observed in the ipsilateral side of the L4 DRG at 20 days after PNI compared to the contralateral side. The mean density of ionized calcium-binding adapter molecule 1 (IBA1)-positive cells was significantly suppressed by intravenous administration of SHED or SHED-CM (Fig. 2c,d).

### SHED attenuates the activation of microglia and astrocytes in the spinal dorsal horn following PNI

**Figure 3:**
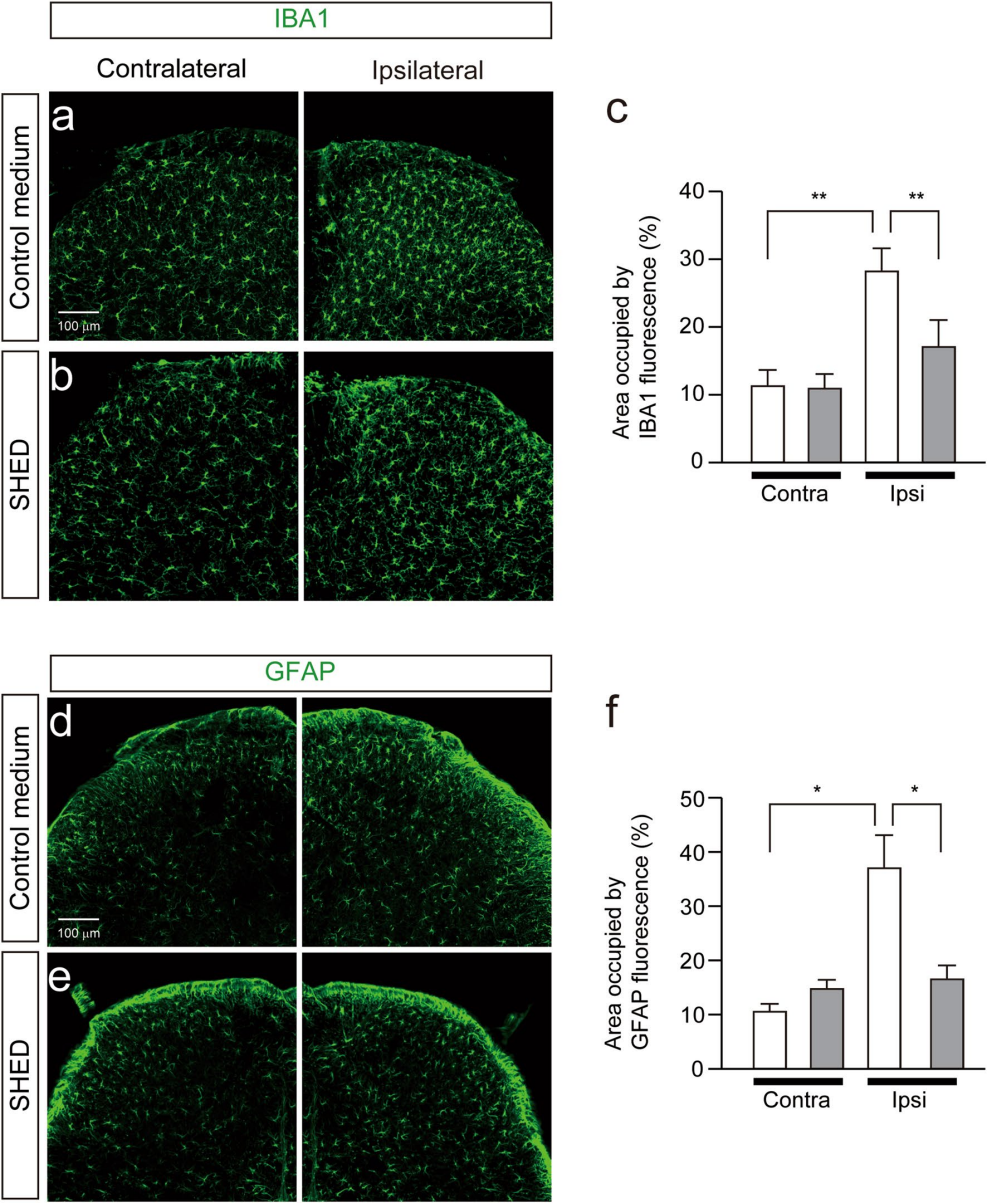
Inhibitory effect of SHED administration on the accumulation of activated microglia and reactive astrocytes in the spinal dorsal horn. (**a**,**b**) The immunofluorescent CLSM images for IBA1 (**a**) and GFAP (**b**) in the spinal dorsal horn 15 days after control medium administration in nerve-injured mice. Scale bar = 100 μm. (**c**) The columns represent the mean area occupied by IBA1 fluorescence in the SDH. Data represent the mean ± SEM. n = 5. Two-way ANOVA Tukey’s test (**p < 0.01). (**d**,**e**) The immunofluorescent CLSM images for IBA1 (**c**) and GFAP (**d**) in the dorsal spinal cord 15 days after SHED administration in nerve-injured mice. Scale bar = 100 μm. (**f**) The columns represent the mean area occupied by IBA1 fluorescence in the SDH. Data represent the mean ± SEM. n = 5. Two-way ANOVA post hoc Tukey’s test (**p < 0.01).

It is well known that microglia and astrocytes are activated following PNI and inhibition of these cells suppresses the development of mechanical allodynia^16,17,23,24^. To analyze the effects of SHED on the activation of spinal microglia and astrocytes, we stained spinal cord slices with anti-IBA1 or glial fibrillary acidic protein (GFAP) antibodies. Both IBA1 and GFAP immunofluorescence was increased in the ipsilateral side compared to the contralateral side of the spinal dorsal horn (SDH) after 20 days of PNI (Fig. 3a–f). In contrast, intravenous administration of SHED markedly inhibited the increment of IBA1 and GFAP immunofluorescence in the ipsilateral side of the spinal dorsal horn (Fig. 3a–f).

### Mechanical allodynia was unaffected by a single intravenous administration of ED-siglec-9 and MCP-1

**Figure 4:**
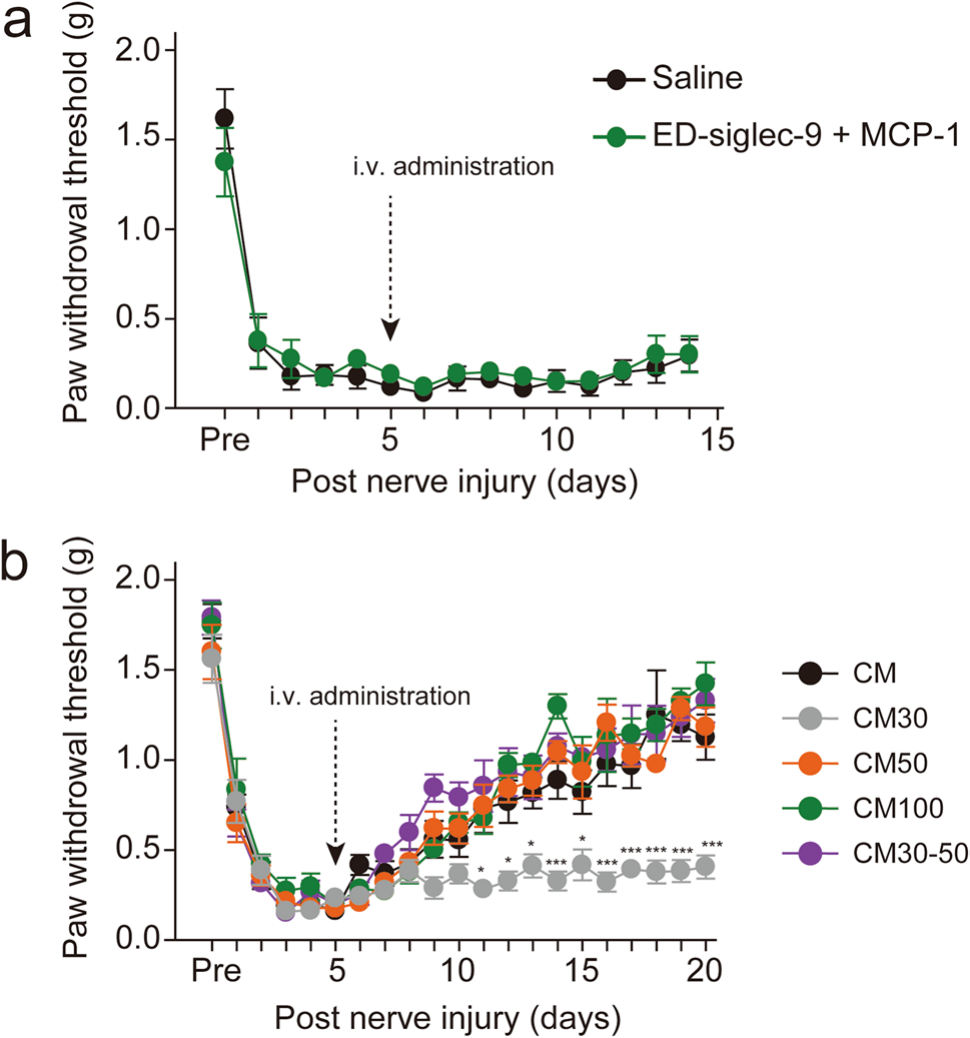
(**a**) The effects of ED-siglec-9 + MCP-1 on the development of mechanical allodynia. Co-administration of ED-siglec-9 + MCP-1 showed no effect of the mean PWT. n = 4 (Saline); n = 5 (ED-siglec-9 + MCP-1), two-way ANOVA repeated measures ANOVA post hoc Bonferroni’s test, not significant, Saline vs. ED-siglec-9 + MCP-1. (**b**) The effects of SHED-CM on the development of mechanical allodynia. Data represents the mean ± SEM. n = 5 (CM); n = 5 (CM30); n = 6 (CM50); n = 5 (CM100); n = 6 (CM30-50), two-way ANOVA repeated measures ANOVA post hoc Dunnet’s test (*p < 0.05, **p < 0.01, ***p < 0.001, CM vs. CM30).

Next, the effects of combined injection of ED-siglec-9 and MCP-1 were examined on the PNI-induced mechanical allodynia, because ED-siglec-9 and MCP-1 synergistically promoted recovery in a rat model of spinal cord injury by altering the macrophage polarity toward the anti-inflammatory M2 state^11^. However, a combined intravenous administration of human recombinant ED-siglec-9 and MCP-1 had no significant effect on the mean PWT (Fig. 4a, saline, n = 4; ED-Siglec-9 + MCP-1, n = 5).

### Effects of a single intravenous administration of filtrated SHED-CM on mechanical allodynia

In order to narrow down the molecular weight range of active components in SHED-CM, SHED-CM was subjected to ultrafiltration using the membrane filters to cut off molecules with a molecular mass larger than 30, 50, and 100 kDa. A single intravenous administration injection of SHED-CM, which cut off molecules with a molecular mass larger than approximately 30 kDa, showed no significant effect on the mean PWT (Fig. 4b, CM30). On the other hand, the administration of SHED-CM, which cut off molecules with a molecular mass larger than approximately 50 or 100 kDa, reversed the established mechanical allodynia at 6 days after injection (Fig. 4b, CM50, CM100). Furthermore, the administration of SHED-CM with a molecular mass between 30 and 50 kDa also reversed the established mechanical allodynia at 6 days after injection (Fig. 4b, CM30-50). These observations suggest that active components present in SHED-CM that exhibit an ant-allodynic effect is heat-stable molecule(s) with the molecular weight ranging from 30 to 50 kDa.

## Discussion

The present study has provided evidence that a single intravenous injection of either SHED or SHED-CM through the left external jugular vein reversed the established mechanical allodynia in mice. Rather surprisingly, the anti-allodynic effects of SHED or SHED-CM persisted throughout the observation period of 20 days. Consistent with the present observations, a single intraspinal administration of SHED-CM loaded in collagen hydrogel showed anti-allodynic effects in the spinal cord injury model in rats^12^. In addition, the mean head withdrawal threshold in rats with infraorbital nerve ligation was significantly increased by a single systemic or local injection of SHED^20^. Accordingly, SHED has a potential therapeutic effect on severe neuropathic pain in animals.

Our histological analyses here showed that a single intravenous injection of SHED or SHED-CM significantly reduced the mean number of AFT3-positive neurons and the accumulation of macrophages in the ipsilateral side of DRG. ATF3 is invariably induced in DRG neurons after PNI^17,22^. Guan et al. found that after PNI, ATF3-expressing DRG neurons expressed colony-stimulating factor (CSF-1) which was transported to the spinal dorsal horn and activated microglia^17^. This evidence implies that ATF3 might regulate the expression of CSF-1 in DRG neurons. Spatiotemporal analysis revealed activation patterns of microglia in the SDH after PNI. Following L4 nerve injury, an increased expression of calcitonin gene-related peptide associated with microglial activation was spread from the L4 toward the L3 and S1 SDH^25^. Thereby, activated spinal microglia in the L3 or L5 SDH potentiate nociceptive information from the hind paw. Activated microglia secrete several molecules including interleukin-18 and complement C1q which activate astrocytes, resulting in the exacerbation of neuropathic pain^26,27^. Considering the above evidence and our results that SHED and SHED-CM suppressed ATF3-expression in the DRG and the activation of microglia and astrocytes, it is conceivable that SHED and SHED-CM alter activated type of microglia and astrocytes into a quiescent phenotype by interrupting the sequential signal relay initiated by ATF3 induction in DRG neurons. More recently, critical involvement of accumulated macrophages in the DRG on maintenance of neuropathic pain has been reported^19^. In fact, SHED and SHED-CM have suppressed the accumulation of macrophages in the DRG following PNI. Through the above-mentioned mechanisms, SHED or SHED-CM are thought to have a long-term anti-allodynic effect by a single intravenous administration. Unfortunately, an intravenous injection of MCP-1 and ED-Siglec-9, which improved spinal cord injury^11^, had no effect on the mechanical allodynia. Future studies will identify the target analgesic molecules. In the present study, there is a gap in the onset of anti-allodynic effects between SHED and SHED-CM treatment. Considering that SHED was selectively accumulated in the damaged DRG, it is conceivable that SHED was anchored in the DRG and released anti-allodynic molecule(s), resulting in the suppression of ATF3 expression in DRG neurons and macrophage accumulation in the DRG.

Despite the anti-allodynic effects of SHED, we did not address how many SHED is anchored in the DRG. We assessed the accumulation of 6-CFDA-labeled SHED in the DRG 3 days after its administration because the fluorescence intensity of 6-CFDA could not maintain for a long time. However, 6-CFDA-labeled SHED selectively migrated in the damaged site but not in the intact site, and anti-allodynic effects of SHED lasted for 20 days. After SHED administration, SHED migrated into the injured but not the intact side of the trigeminal ganglion after infraorbital nerve injury (a model of orofacial neuropathic pain) in rats^20^. Furthermore, the anti-allodynic effects of SHED lasted for 8 weeks^20^. Accordingly, long-lasting anti-allodynic effects might be observed in our case. Following the migration into the damaged tissue, SHED can differentiate into functional cells^2,28^. In addition to the factors secreted by SHED, it is also possible that the differentiation of SHED is responsible for the anti-allodynic effect. In the future, we need to analyze the profile of migrated SHED in the injured side of DRG.

The close examination revealed that the molecular weight of proteins component(s) responsible for the anti-allodynic effect of SHED-CM ranged from 30 to 50 kDa. SHED-CM contains growth factors, proteases, and immune modulators with the molecular mass of 30–50 kDa, including vascular endothelial growth factor (VEGF), placental growth factor, matrix metalloproteinase-3, marapsin, follistatin, osteopontin, and CD40^11^. Among them, VEGF is the most likely mediator of the anti-allodynic effect exerted by SHED-CM, because VEGF has an important neuroprotective effect during neuropathic conditions^29^ and anti-nociceptive effects in experimental painful conditions^29,30^. However, VEGF has been also shown to induce pronociceptive effects^31^. Therefore, it is likely to consider that some of these protein factors may synergistically cause protective and/or anti-inflammatory activity to induce an anti-allodynic effect. We found a strong anti-allodynic activity SHED-CM ranged from 30 to 50 kDa. However, the anti-allodynic effect of SHED-CM was also observed when the molecular weight was cut above 100 kDa. From this observation, the possibility of the existence of analgesic factors in the range of 50 to 100 kDa cannot be ruled out.

The proliferation rate of stem cells from SHED is significantly higher than that of BMMSCs and dental pulp stem cells^2,32,33^. SHED can be easily obtained, without the need for invasive procedures and thus represent a large source of stem cells for potential clinical application. Therefore, SHED may have advantages over BMMSCs in pain control and potentially become an alternate and efficacious treatment for neuropathic pain. Furthermore, considering the short-lasting anti-nociceptive effect of gabapentin, a first-line agent for the treatment of neuropathic pain, the long-lasting property of SHED-CM has greater clinical advantages. Further studies will be needed to identify and characterize protein factors responsible for the anti-allodynic effect of SHED-CM.

## Materials and methods

### Isolation and culture of SHED and preparation of conditioned medium from SHED

Experiments using human samples were reviewed and approved by the Kyushu University Institutional Review Board for Human Genome/Gene Research (Protocol Number: 677-00) and were conducted in accordance with the Declaration of Helsinki. Informed consent was obtained from the patients’ guardians. Human exfoliated deciduous teeth were collected as discarded biological/clinical samples from children (5–7-year-old) at the Department of Pediatrics of Kyushu University Hospital. The SHED was isolated as previously described^34^ and cultured in Alpha Modification of Eagle's Medium (Sigma-Aldrich, MO, USA) containing 15% fetal bovine serum (Sigma-Aldrich), 100 μM l-ascorbic acid 2-phosphate (Wako Pure Chemical Industries, Osaka, Japan), 2 mM l-glutamine (Thermo Fisher Scientific, MA, USA), 250 μg/ml fungizone (Thermo Fisher Scientific), 100 U/ml penicillin, and 100 μg/ml streptomycin (Thermo Fisher Scientific), at 37 °C, in an atmosphere containing 5% CO_2_. The cells were used for further experiments as a heterogeneous cell population. In some experiments, SHED was incubated with 6-CFDA (100 nM, 15 min, Thermo Fisher Scientific), and then injected through the external jugular vein as described below.

To obtain SHED-CM, the cells were cultured to subconfluency and incubated in 10 ml of serum-free medium for 48 h. The supernatant was collected and centrifuged at 500 rpm for 5 min. The supernatant was recentrifuged at 3000 rpm for 3 min and the sample was collected. SHED-CM was subjected to a fivefold concentration (5× SHED-CM) using Amicon Ultra (Merck Millipore, MA, USA) according to the manufacture's protocol. To inactivate SHED-CM, SHED-CM was heated at 95 °C for 5 min. Fractionation of SHED-CM based on molecular size was conducted using Amicon Ultra 30 k, 50 k, or 100 k according to the manufactures' protocol. Serum-free alpha Modification of Eagle's Medium was used as the control medium.

### Animals

Male C57BL/6J mice (8–10 weeks old) were purchased from CLEA Japan, Inc. The mice were maintained in a 12 h light/dark cycle (light beginning at 08:00) at 22 ± 1 °C ambient temperature with food and water provided ad libitum. All mice were handled daily for 5 days prior to the initiation of the experiment to minimize their stress reactions to manipulation. Animal protocols were approved by the Experimentation committee in Kyushu University (Protocol numbers: A28-201 and A30-062). The experiments complied with the ethical guidelines of the International Association for the Study of Pain^35^. Animal studies are reported in compliance with the ARRIVE (Animal Research: Reporting of In Vivo Experiments) guidelines.

### Surgical procedure

For the neuropathic pain model, PNI was made by the transection of the L4 spinal nerve without injury to the adjacent nerve under 2% isoflurane inhalation according to the method as described previously^16^.

For the intravenous administration, the left external jugular vein was exposed with blunt dissection. Intrajuglar injection was made using a 31-gauge needle after passing through the pectoral muscle**.** SHED (2 × 10^5^ cells/100 μl serum-free medium), boiled SHED (100 μl), 5 × SHED-CM (100 μl), filtered SHED-CM (100 μl), or human recombinant ED-siglec-9 and MCP-1 (100 ng/ml in each) were intrajuglarly delivered 5 days after PNI. 6-CFDA-labeled SHED (2 × 10^5^ cells/100 μl serum-free medium) was intravenously injected 2 days after PNI.

### Behavioral testing

The mice were examined for mechanical hypersensitivity of the hind paw following PNI. All mice were habituated to the testing environment for 3 days and were examined for mechanical allodynia according to the previous method^16^. The room temperature remained stable at 22 ± 1 °C. The mice were placed in an acrylic cylinder (6 cm diameter) with wire mesh floors and allowed to habituate to the testing environment for 1 h. The calibrated von Frey filaments (0.02, 0.04, 0.07, 0.16, 0.4, 0.6, 1.0, 1.4, and 2.0 g; North Coast Medical, Inc., CA, USA) were applied to the midplantar surface of the hind paw. The paw withdrawal thresholds were calculated using the up-down method^36^. Behavioral tests were performed once a daily for 20 days.

### Tissue preparation and immunohistochemistry

The mice were euthanized with sodium pentobarbital (200 mg/kg, i.p.) after cessation of the behavioral analyses. Then, the mice were perfused transcardially with 0.01 M phosphate buffer saline (PBS; Sigma-Aldrich), pH 7.4, followed by 4% paraformaldehyde (PFA) in 0.1 M PB, 5 and 20 days after PNI. The L4 spinal segments and L3, L4, and L5 DRG were excised and further fixed with 4% PFA overnight at 4 °C. They were further incubated with 30% sucrose (Sigma-Aldrich) for 2 days at 4 °C to protect cryolesion. The sections were made at a thickness of 40 μm for the spinal cord and 10 μm for the DRG by a CM1860 cryomicrotome (Leica Microsystems, Inc., Wetzlar, Germany). Blocking was performed by 1% normal donkey serum (Jackson ImmunoResearch Laboratories, Inc.), 1% BSA (Sigma-Aldrich), and 0.4% Triton X-100 (Sigma-Aldrich) in PBS for 1 h. The DRG and spinal slices were incubated with rabbit polyclonal anti-ATF3 antibody (1:1000; Cat.No. sc-188, Santa Cruz, CA, USA) overnight at 4 °C. The spinal slices were incubated with rabbit polyclonal anti-IBA1 antibody (1:5000; Cat.No.019-19741, Fujifilm Wako Pure Chemical Corporation) or rabbit polyclonal anti-GFAP antibody (1:5000; Cat. No. Z0334; Dako, Glostrup, Denmark) for 2 days at 4 °C. Following washing with PBS for three times, the slices were incubated with donkey anti-rabbit IgG conjugated with Cy3 or Alexa488 (1:400 in each; Jackson ImmunoResearch Laboratories, Inc., PA, USA) and NeuroTrace 435/455 Blue Fluorescent Nissl Stain (1:1000; Cat. No. N21479, Thermo Fisher Scientific) for 2 h at 4 °C, and then mounted in Vectashield (Vector Laboratories, Inc.). The images were acquired on a Nikon C2 scanning confocal microscope using a 20× objective lens (NA 0.75; Nikon Corporation, Tokyo, Japan). 6-CFDA-labeled SHED and ATF3-positive and Nissl-positive DRG neurons and IBA1-positive cells in the images (image size: 460 × 460 μm^2^) were counted by Image J software (version 1.53k) plugin Cell count (NIH; http://rsbweb.nih.gov/ij/). The signal was defined as the fluorescence intensity that was five times greater than the noise intensity. The ratio of ATF3-positive neurons in total DRG neurons was calculated. The area occupied by IBA1 fluorescence in the DRG or IBA1 or GFAP immunofluorescence in the SDH was measured by Image J following making binarized images from original images. Three sections from one mouse were stained, and the mean value was taken as the value for one mouse.

### Statistical analyses

The data are represented as the mean ± standard error of the mean (SEM). Data normality was assessed by the Shapiro–Wilk test. Statistical analyses of the results were performed with one-way analysis of variance (ANOVA) post hoc Tukey test, and two-way repeated-measures ANOVA post hoc Bonferroni’s or Dunnet’s test using the GraphPad Prism9 (GraphPad Software, Inc., CA, USA) software package. *p* < 0.05 was considered to indicate a statistically significant difference. Graphical images were made by Prism 9 and Illustrator 2021 (Adobe Inc., CA, USA).

### Ethical approval

Experiments using human samples and animals were approved by Institutional Review Board for Human Genome/Gene Research (Protocol Number: 677-00) and the Experimentation Committee of Kyushu University (Protocol numbers: A28-201 and A30-062), respectively.

## Data Availability

The data used in this study are available from the corresponding authors upon reasonable request.
